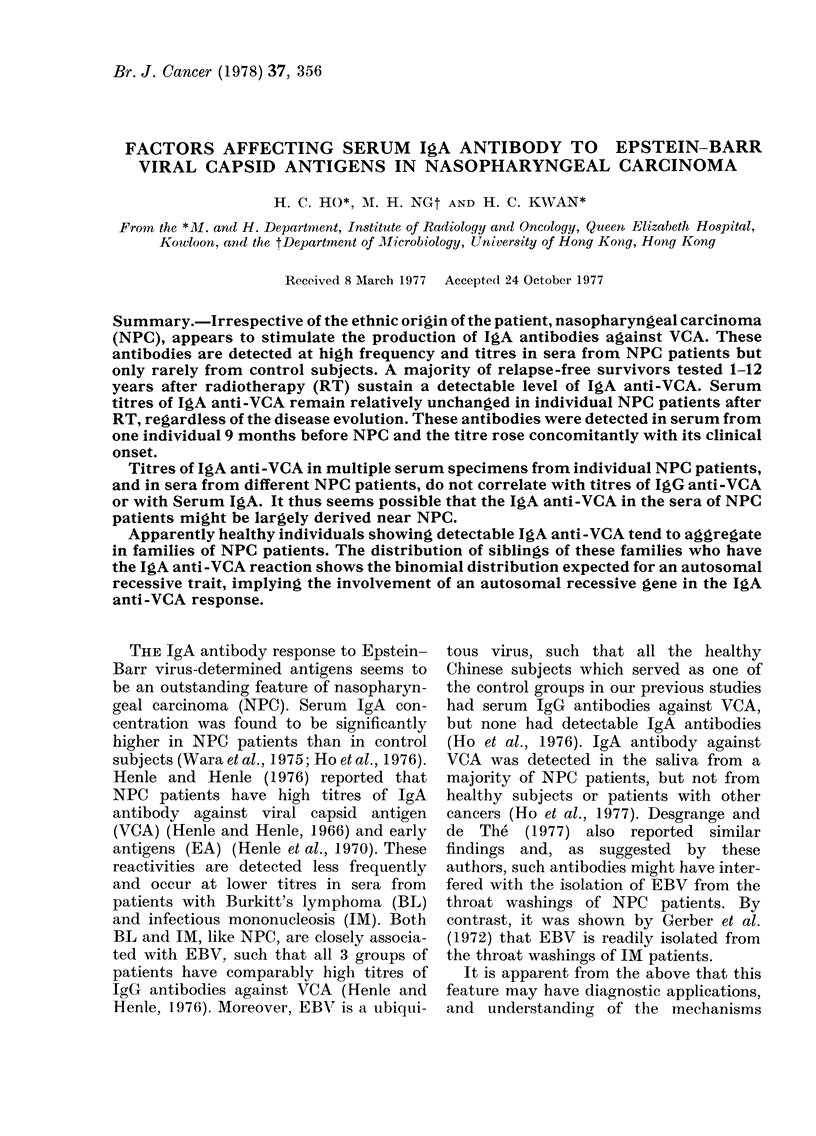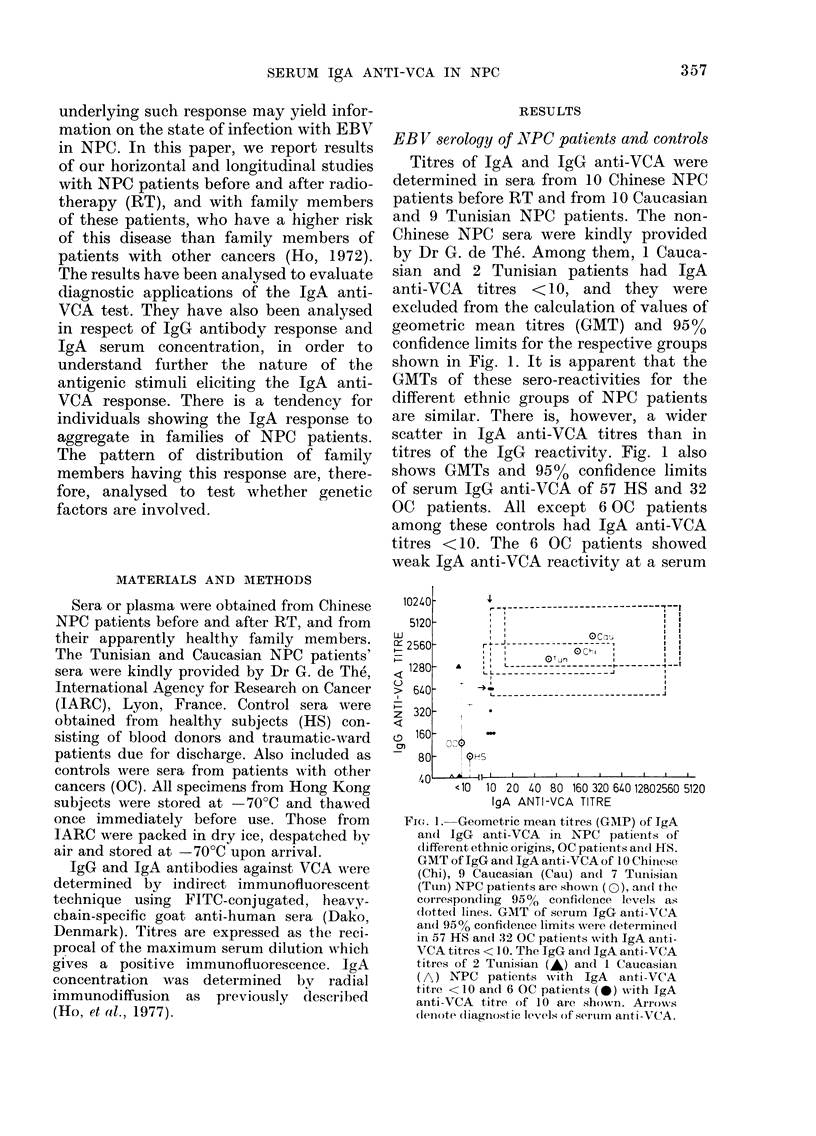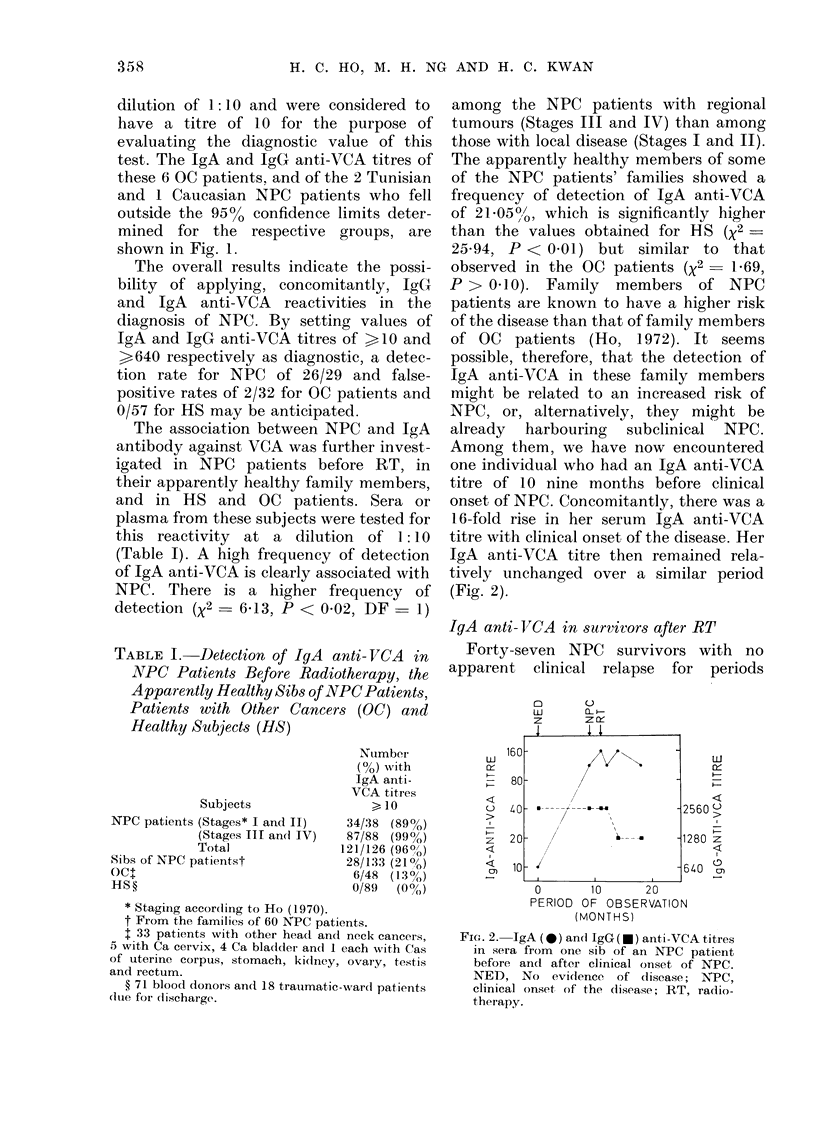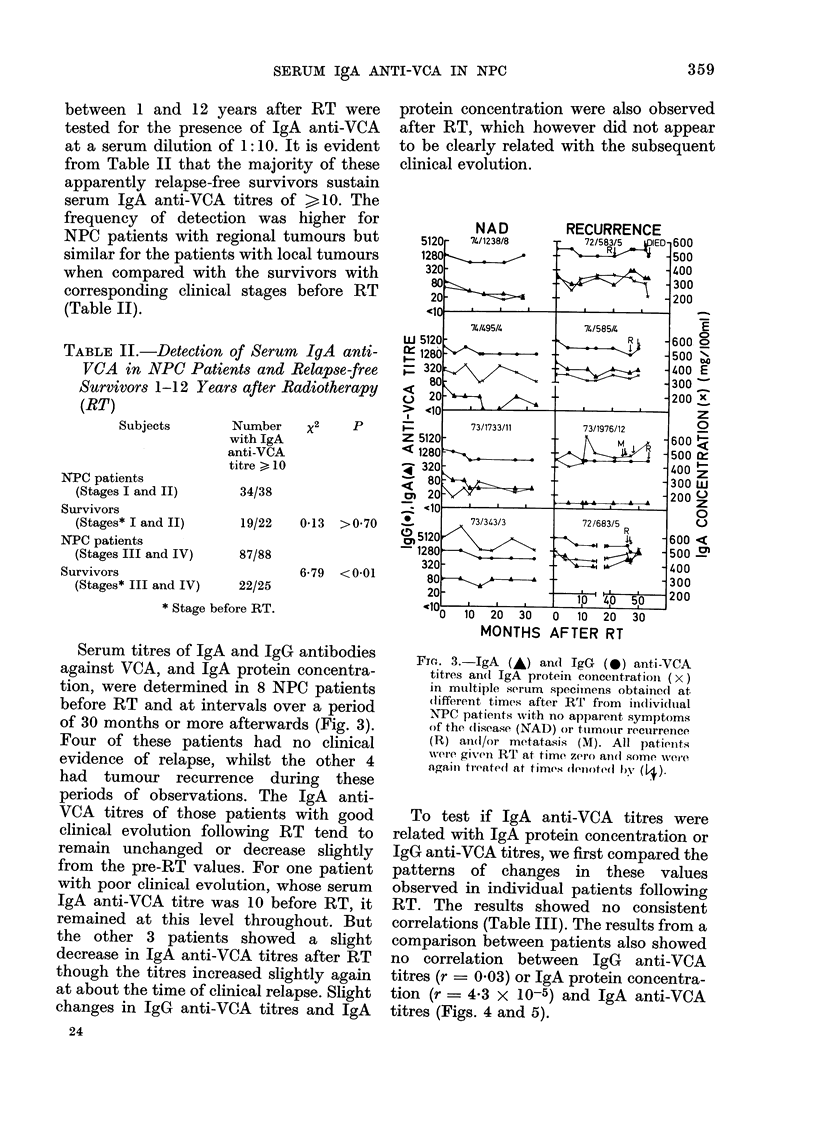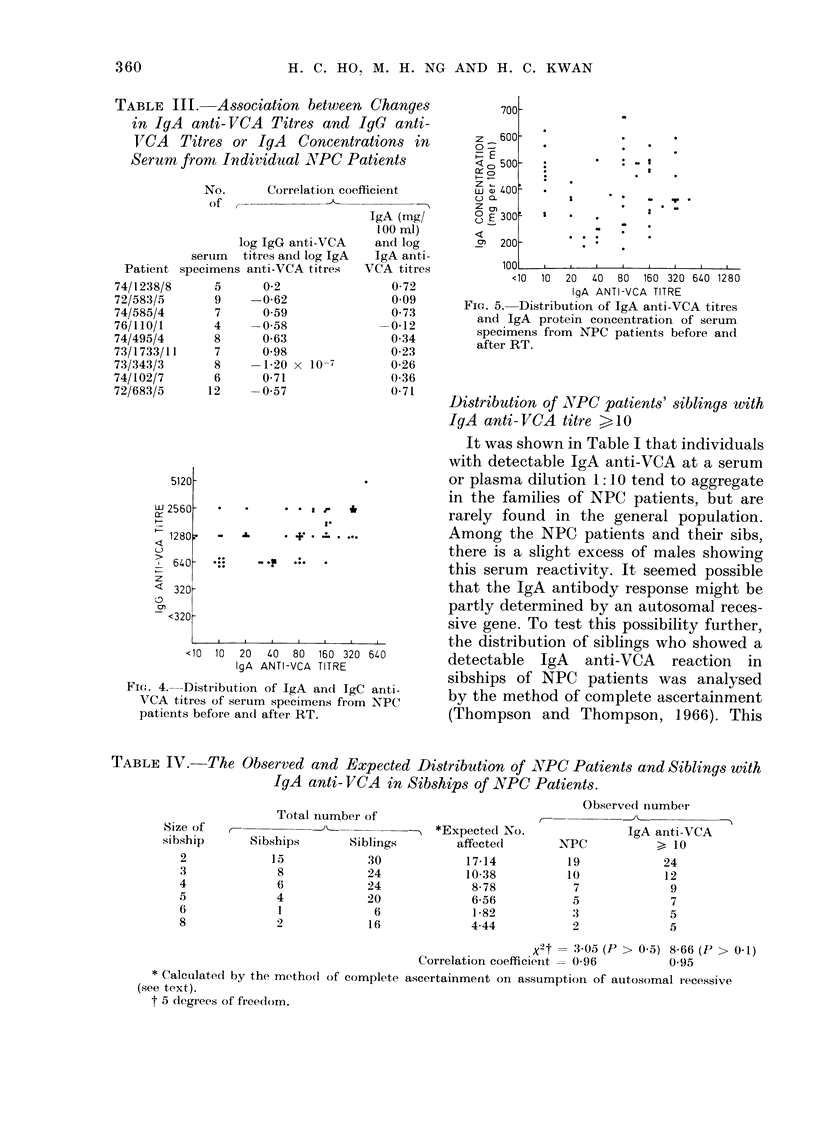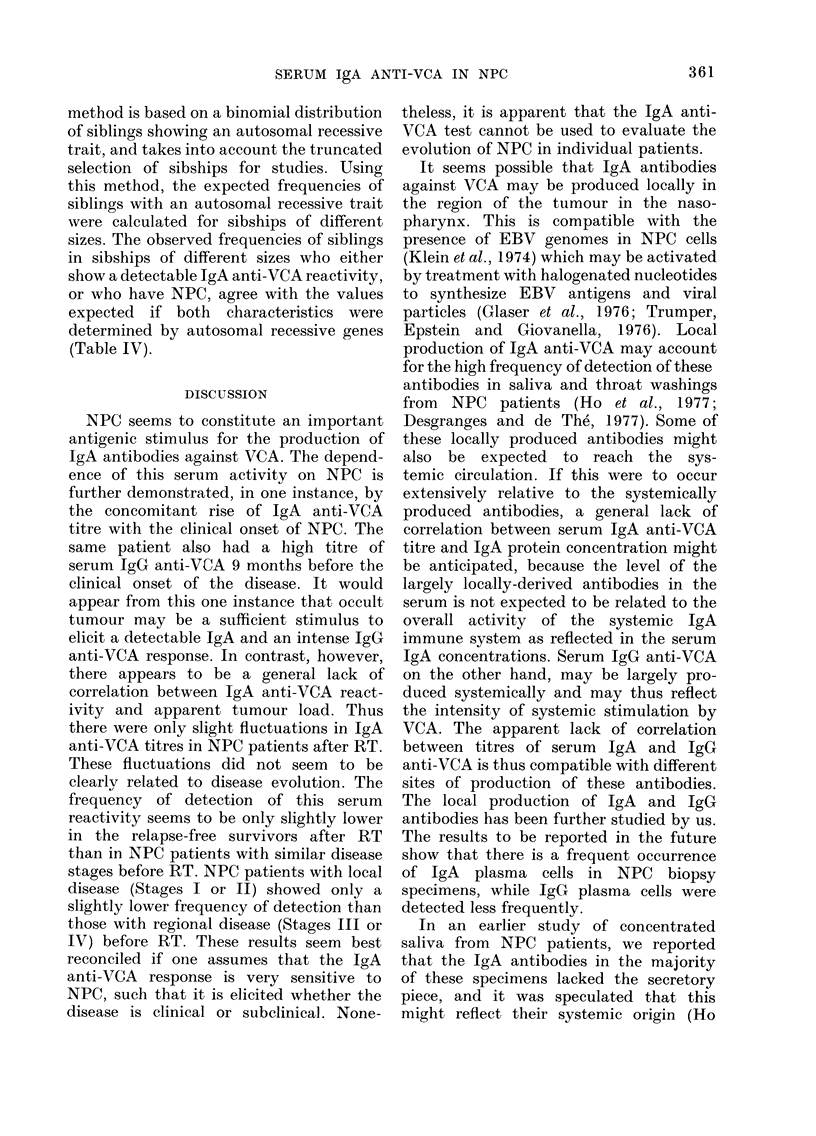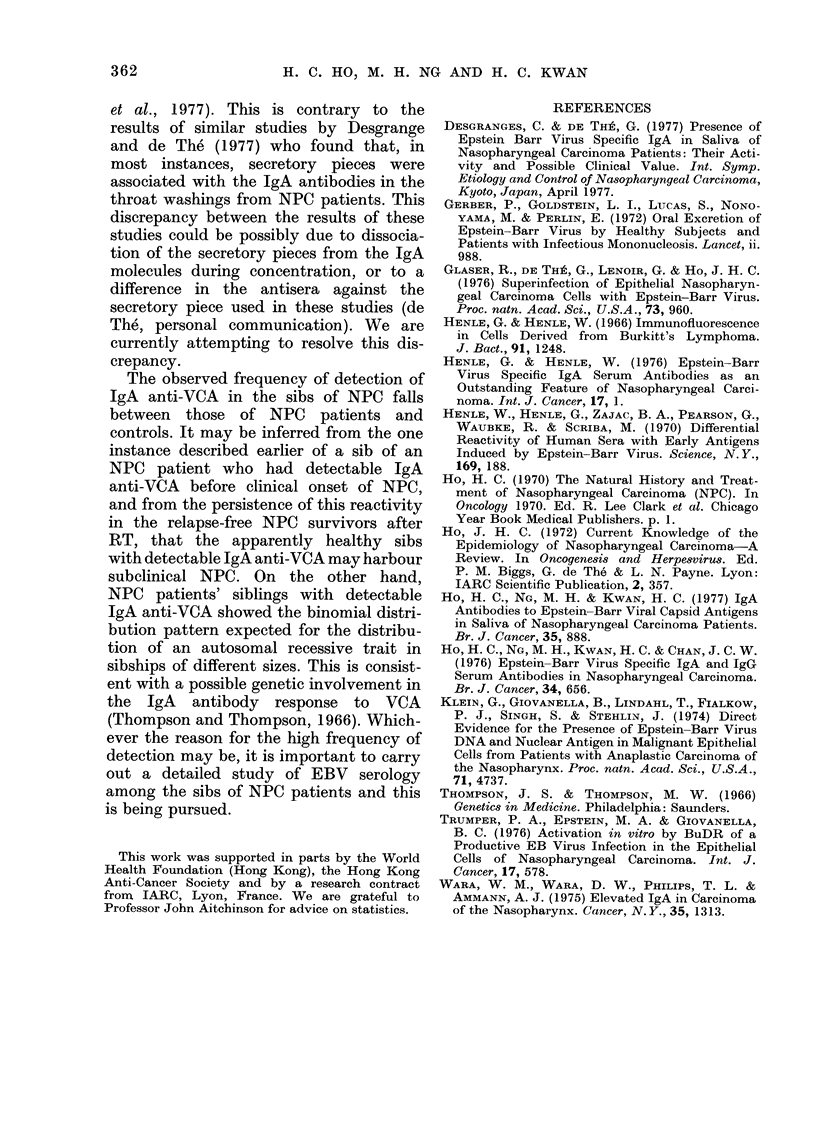# Factors affecting serum IgA antibody to Epstein-Barr viral capsid antigens in nasopharyngeal carcinoma.

**DOI:** 10.1038/bjc.1978.54

**Published:** 1978-03

**Authors:** H. C. Ho, M. H. Ng, H. C. Kwan

## Abstract

Irrespective of the ethnic origin of the patient, nasopharyngeal carcinoma (NPC), appears to stimulate the production of IgA antibodies against VCA. These antibodies are detected at high frequency and titres in sera from NPC patients but only rarely from control subjects. A majority of relapse-free survivors tested 1-12 years after radiotherapy (RT) sustain a detectable level of IgA anti-VCA. Serum titres of IgA anti-VCA remain relatively unchaged in individual NPC patients after RT, regardless of the disease evolution. These antibodies were detected in serum from one individual 9 months before NPC and the titre rose concomitantly with its clinical onset. Titres of IgA anti-VCA in multiple serum specimens from individual NPC patients, and in sera from different NPC patients, do not correlate with titres of IgG anti-VCA or with Serum IgA. It thus seems possible that the IgA anti-VCA in the sera of NPC patients might be largely derived near NPC. Apparently healthy individuals showing detectable IgA anti-VCA tend to aggregate in families of NPC patients. The distribution of siblings of these families who have the IgA anti-VCA reaction shows the binomial distribution expected for an autosomal recessive trait, implying the involvement of an autosomal recessive gene in the IgA anti-VCA response.


*Br. J. Cancer* (1978) **37**, 356

### FACTORS AFFECTING SERUM IgA ANTIBODY TO EPSTEIN–BARR VIRAL CAPSID ANTIGENS IN NASOPHARYNGEAL CARCINOMA

H. C. HO*, M. H. NG† AND H. C. KWAN*

*From the *M. and H. Department, Institute of Radiology and Oncology, Queen Elizabeth Hospital, Kowloon, and the †Department of Microbiology, University of Hong Kong, Hong Kong*

Received 8 March 1977 Accepted 24 October 1977

**Summary.—Irrespective of the ethnic origin of the patient, nasopharyngeal carcinoma (NPC), appears to stimulate the production of IgA antibodies against VCA. These antibodies are detected at high frequency and titres in sera from NPC patients but only rarely from control subjects. A majority of relapse-free survivors tested 1–12 years after radiotherapy (RT) sustain a detectable level of IgA anti-VCA. Serum titres of IgA anti-VCA remain relatively unchanged in individual NPC patients after RT, regardless of the disease evolution. These antibodies were detected in serum from one individual 9 months before NPC and the titre rose concomitantly with its clinical onset.**

**Titres of IgA anti-VCA in multiple serum specimens from individual NPC patients, and in sera from different NPC patients, do not correlate with titres of IgG anti-VCA or with Serum IgA. It thus seems possible that the IgA anti-VCA in the sera of NPC patients might be largely derived near NPC.**

**Apparently healthy individuals showing detectable IgA anti-VCA tend to aggregate in families of NPC patients. The distribution of siblings of these families who have the IgA anti-VCA reaction shows the binomial distribution expected for an autosomal recessive trait, implying the involvement of an autosomal recessive gene in the IgA anti-VCA response.**

THE IgA antibody response to Epstein–Barr virus-determined antigens seems to be an outstanding feature of nasopharyngeal carcinoma (NPC). Serum IgA concentration was found to be significantly higher in NPC patients than in control subjects (Wara *et al.*, 1975; Ho *et al.*, 1976). Henle and Henle (1976) reported that NPC patients have high titres of IgA antibody against viral capsid antigen (VCA) (Henle and Henle, 1966) and early antigens (EA) (Henle *et al.*, 1970). These reactivities are detected less frequently and occur at lower titres in sera from patients with Burkitt's lymphoma (BL) and infectious mononucleosis (IM). Both BL and IM, like NPC, are closely associated with EBV, such that all 3 groups of patients have comparably high titres of IgG antibodies against VCA (Henle and Henle, 1976). Moreover, EBV is a ubiquitous virus, such that all the healthy Chinese subjects which served as one of the control groups in our previous studies had serum IgG antibodies against VCA, but none had detectable IgA antibodies (Ho *et al.*, 1976). IgA antibody against VCA was detected in the saliva from a majority of NPC patients, but not from healthy subjects or patients with other cancers (Ho *et al.*, 1977). Desgrange and de Thé (1977) also reported similar findings and, as suggested by these authors, such antibodies might have interfered with the isolation of EBV from the throat washings of NPC patients. By contrast, it was shown by Gerber *et al.* (1972) that EBV is readily isolated from the throat washings of IM patients.

It is apparent from the above that this feature may have diagnostic applications, and understanding of the mechanisms underlying such response may yield information on the state of infection with EBV in NPC. In this paper, we report results of our horizontal and longitudinal studies with NPC patients before and after radiotherapy (RT), and with family members of these patients, who have a higher risk of this disease than family members of patients with other cancers (Ho, 1972). The results have been analysed to evaluate diagnostic applications of the IgA anti-VCA test. They have also been analysed in respect of IgG antibody response and IgA serum concentration, in order to understand further the nature of the antigenic stimuli eliciting the IgA anti-VCA response. There is a tendency for individuals showing the IgA response to aggregate in families of NPC patients. The pattern of distribution of family members having this response are, therefore, analysed to test whether genetic factors are involved.

### MATERIALS AND METHODS

Sera or plasma were obtained from Chinese NPC patients before and after RT, and from their apparently healthy family members. The Tunisian and Caucasian NPC patients' sera were kindly provided by Dr G. de Thé, International Agency for Research on Cancer (IARC), Lyon, France. Control sera were obtained from healthy subjects (HS) consisting of blood donors and traumatic-ward patients due for discharge. Also included as controls were sera from patients with other cancers (OC). All specimens from Hong Kong subjects were stored at $-70°C$ and thawed once immediately before use. Those from IARC were packed in dry ice, despatched by air and stored at $-70°C$ upon arrival.

IgG and IgA antibodies against VCA were determined by indirect immunofluorescent technique using FITC-conjugated, heavy-chain-specific goat anti-human sera (Dako, Denmark). Titres are expressed as the reciprocal of the maximum serum dilution which gives a positive immunofluorescence. IgA concentration was determined by radial immunodiffusion as previously described (Ho, *et al.*, 1977).

### RESULTS

### *EBV serology of NPC patients and controls*

Titres of IgA and IgG anti-VCA were determined in sera from 10 Chinese NPC patients before RT and from 10 Caucasian and 9 Tunisian NPC patients. The non-Chinese NPC sera were kindly provided by Dr G. de Thé. Among them, 1 Caucasian and 2 Tunisian patients had IgA anti-VCA titres $<10$, and they were excluded from the calculation of values of geometric mean titres (GMT) and 95% confidence limits for the respective groups shown in Fig. 1. It is apparent that the GMTs of these sero-reactivities for the different ethnic groups of NPC patients are similar. There is, however, a wider scatter in IgA anti-VCA titres than in titres of the IgG reactivity. Fig. 1 also shows GMTs and 95% confidence limits of serum IgG anti-VCA of 57 HS and 32 OC patients. All except 6 OC patients among these controls had IgA anti-VCA titres $<10$. The 6 OC patients showed weak IgA anti-VCA reactivity at a serum

FIG. 1.—Geometric mean titres (GMP) of IgA and IgG anti-VCA in NPC patients of different ethnic origins, OC patients and HS. GMT of IgG and IgA anti-VCA of 10 Chinese (Chi), 9 Caucasian (Cau) and 7 Tunisian (Tun) NPC patients are shown (○), and the corresponding 95% confidence levels as dotted lines. GMT of serum IgG anti-VCA and 95% confidence limits were determined in 57 HS and 32 OC patients with IgA anti-VCA titres $<10$. The IgG and IgA anti-VCA titres of 2 Tunisian (▲) and 1 Caucasian (△) NPC patients with IgA anti-VCA titre $<10$ and 6 OC patients (●) with IgA anti-VCA titre of 10 are shown. Arrows denote diagnostic levels of serum anti-VCA.

dilution of 1:10 and were considered to have a titre of 10 for the purpose of evaluating the diagnostic value of this test. The IgA and IgG anti-VCA titres of these 6 OC patients, and of the 2 Tunisian and 1 Caucasian NPC patients who fell outside the 95% confidence limits determined for the respective groups, are shown in Fig. 1.

The overall results indicate the possibility of applying, concomitantly, IgG and IgA anti-VCA reactivities in the diagnosis of NPC. By setting values of IgA and IgG anti-VCA titres of $\geqslant 10$ and $\geqslant 640$ respectively as diagnostic, a detection rate for NPC of 26/29 and false-positive rates of 2/32 for OC patients and 0/57 for HS may be anticipated.

The association between NPC and IgA antibody against VCA was further investigated in NPC patients before RT, in their apparently healthy family members, and in HS and OC patients. Sera or plasma from these subjects were tested for this reactivity at a dilution of 1:10 (Table I). A high frequency of detection of IgA anti-VCA is clearly associated with NPC. There is a higher frequency of detection ($\chi^2 = 6{\cdot}13$, $P < 0{\cdot}02$, DF $= 1$) among the NPC patients with regional tumours (Stages III and IV) than among those with local disease (Stages I and II). The apparently healthy members of some of the NPC patients' families showed a frequency of detection of IgA anti-VCA of 21·05%, which is significantly higher than the values obtained for HS ($\chi^2 = 25{\cdot}94$, $P < 0{\cdot}01$) but similar to that observed in the OC patients ($\chi^2 = 1{\cdot}69$, $P > 0{\cdot}10$). Family members of NPC patients are known to have a higher risk of the disease than that of family members of OC patients (Ho, 1972). It seems possible, therefore, that the detection of IgA anti-VCA in these family members might be related to an increased risk of NPC, or, alternatively, they might be already harbouring subclinical NPC. Among them, we have now encountered one individual who had an IgA anti-VCA titre of 10 nine months before clinical onset of NPC. Concomitantly, there was a 16-fold rise in her serum IgA anti-VCA titre with clinical onset of the disease. Her IgA anti-VCA titre then remained relatively unchanged over a similar period (Fig. 2).

TABLE I.—*Detection of IgA anti-VCA in NPC Patients Before Radiotherapy, the Apparently Healthy Sibs of NPC Patients, Patients with Other Cancers (OC) and Healthy Subjects (HS)*

| Subjects | Number (%) with IgA anti-VCA titres $\geqslant 10$ |
|---|---|
| NPC patients (Stages* I and II) | 34/38 (89%) |
| (Stages III and IV) | 87/88 (99%) |
| Total | 121/126 (96%) |
| Sibs of NPC patients† | 28/133 (21%) |
| OC‡ | 6/48 (13%) |
| HS§ | 0/89 (0%) |

* Staging according to Ho (1970).

† From the families of 60 NPC patients.

‡ 33 patients with other head and neck cancers, 5 with Ca cervix, 4 Ca bladder and 1 each with Cas of uterine corpus, stomach, kidney, ovary, testis and rectum.

§ 71 blood donors and 18 traumatic-ward patients due for discharge.

FIG. 2.—IgA (●) and IgG (■) anti-VCA titres in sera from one sib of an NPC patient before and after clinical onset of NPC. NED, No evidence of disease; NPC, clinical onset of the disease; RT, radiotherapy.

### *IgA anti-VCA in survivors after RT*

Forty-seven NPC survivors with no apparent clinical relapse for periods between 1 and 12 years after RT were tested for the presence of IgA anti-VCA at a serum dilution of 1:10. It is evident from Table II that the majority of these apparently relapse-free survivors sustain serum IgA anti-VCA titres of ⩾10. The frequency of detection was higher for NPC patients with regional tumours but similar for the patients with local tumours when compared with the survivors with corresponding clinical stages before RT (Table II).

TABLE II.—*Detection of Serum IgA anti-VCA in NPC Patients and Relapse-free Survivors* 1–12 *Years after Radiotherapy* (*RT*)

| Subjects | Number with IgA anti-VCA titre ⩾10 | $\chi^2$ | *P* |
|---|---|---|---|
| NPC patients (Stages I and II) | 34/38 | | |
| Survivors (Stages* I and II) | 19/22 | 0·13 | >0·70 |
| NPC patients (Stages III and IV) | 87/88 | | |
| Survivors (Stages* III and IV) | 22/25 | 6·79 | <0·01 |

* Stage before RT.

Serum titres of IgA and IgG antibodies against VCA, and IgA protein concentration, were determined in 8 NPC patients before RT and at intervals over a period of 30 months or more afterwards (Fig. 3). Four of these patients had no clinical evidence of relapse, whilst the other 4 had tumour recurrence during these periods of observations. The IgA anti-VCA titres of those patients with good clinical evolution following RT tend to remain unchanged or decrease slightly from the pre-RT values. For one patient with poor clinical evolution, whose serum IgA anti-VCA titre was 10 before RT, it remained at this level throughout. But the other 3 patients showed a slight decrease in IgA anti-VCA titres after RT though the titres increased slightly again at about the time of clinical relapse. Slight changes in IgG anti-VCA titres and IgA protein concentration were also observed after RT, which however did not appear to be clearly related with the subsequent clinical evolution.

FIG. 3.—IgA (▲) and IgG (●) anti-VCA titres and IgA protein concentration (×) in multiple serum specimens obtained at different times after RT from individual NPC patients with no apparent symptoms of the disease (NAD) or tumour recurrence (R) and/or metatasis (M). All patients were given RT at time zero and some were again treated at times denoted by (↓).

To test if IgA anti-VCA titres were related with IgA protein concentration or IgG anti-VCA titres, we first compared the patterns of changes in these values observed in individual patients following RT. The results showed no consistent correlations (Table III). The results from a comparison between patients also showed no correlation between IgG anti-VCA titres ($r = 0{\cdot}03$) or IgA protein concentration ($r = 4{\cdot}3 \times 10^{-5}$) and IgA anti-VCA titres (Figs. 4 and 5).

TABLE III.—*Association between Changes in IgA anti-VCA Titres and IgG anti-VCA Titres or IgA Concentrations in Serum from Individual NPC Patients*

| Patient | No. of serum specimens | Correlation coefficient: log IgG anti-VCA titres and log IgA anti-VCA titres | Correlation coefficient: IgA (mg/100 ml) and log IgA anti-VCA titres |
|---|---|---|---|
| 74/1238/8 | 5 | 0·2 | 0·72 |
| 72/583/5 | 9 | −0·62 | 0·09 |
| 74/585/4 | 7 | 0·59 | 0·73 |
| 76/110/1 | 4 | −0·58 | −0·12 |
| 74/495/4 | 8 | 0·63 | 0·34 |
| 73/1733/11 | 7 | 0·98 | 0·23 |
| 73/343/3 | 8 | $-1{\cdot}20 \times 10^{-7}$ | 0·26 |
| 74/102/7 | 6 | 0·71 | 0·36 |
| 72/683/5 | 12 | −0·57 | 0·71 |

FIG. 4.—Distribution of IgA and IgC anti-VCA titres of serum specimens from NPC patients before and after RT.

FIG. 5.—Distribution of IgA anti-VCA titres and IgA protein concentration of serum specimens from NPC patients before and after RT.

*Distribution of NPC patients' siblings with IgA anti-VCA titre ⩾10*

It was shown in Table I that individuals with detectable IgA anti-VCA at a serum or plasma dilution 1:10 tend to aggregate in the families of NPC patients, but are rarely found in the general population. Among the NPC patients and their sibs, there is a slight excess of males showing this serum reactivity. It seemed possible that the IgA antibody response might be partly determined by an autosomal recessive gene. To test this possibility further, the distribution of siblings who showed a detectable IgA anti-VCA reaction in sibships of NPC patients was analysed by the method of complete ascertainment (Thompson and Thompson, 1966). This

TABLE IV.—*The Observed and Expected Distribution of NPC Patients and Siblings with IgA anti-VCA in Sibships of NPC Patients.*

| Size of sibship | Total number of Sibships | Total number of Siblings | *Expected No. affected | Observed number NPC | Observed number IgA anti-VCA ⩾ 10 |
|---|---|---|---|---|---|
| 2 | 15 | 30 | 17·14 | 19 | 24 |
| 3 | 8 | 24 | 10·38 | 10 | 12 |
| 4 | 6 | 24 | 8·78 | 7 | 9 |
| 5 | 4 | 20 | 6·56 | 5 | 7 |
| 6 | 1 | 6 | 1·82 | 3 | 5 |
| 8 | 2 | 16 | 4·44 | 2 | 5 |
| | | | $\chi^2$† = | 3·05 ($P$ > 0·5) | 8·66 ($P$ > 0·1) |
| | | | Correlation coefficient = | 0·96 | 0·95 |

* Calculated by the method of complete ascertainment on assumption of autosomal recessive (see text).

† 5 degrees of freedom.

method is based on a binomial distribution of siblings showing an autosomal recessive trait, and takes into account the truncated selection of sibships for studies. Using this method, the expected frequencies of siblings with an autosomal recessive trait were calculated for sibships of different sizes. The observed frequencies of siblings in sibships of different sizes who either show a detectable IgA anti-VCA reactivity, or who have NPC, agree with the values expected if both characteristics were determined by autosomal recessive genes (Table IV).

### DISCUSSION

NPC seems to constitute an important antigenic stimulus for the production of IgA antibodies against VCA. The dependence of this serum activity on NPC is further demonstrated, in one instance, by the concomitant rise of IgA anti-VCA titre with the clinical onset of NPC. The same patient also had a high titre of serum IgG anti-VCA 9 months before the clinical onset of the disease. It would appear from this one instance that occult tumour may be a sufficient stimulus to elicit a detectable IgA and an intense IgG anti-VCA response. In contrast, however, there appears to be a general lack of correlation between IgA anti-VCA reactivity and apparent tumour load. Thus there were only slight fluctuations in IgA anti-VCA titres in NPC patients after RT. These fluctuations did not seem to be clearly related to disease evolution. The frequency of detection of this serum reactivity seems to be only slightly lower in the relapse-free survivors after RT than in NPC patients with similar disease stages before RT. NPC patients with local disease (Stages I or II) showed only a slightly lower frequency of detection than those with regional disease (Stages III or IV) before RT. These results seem best reconciled if one assumes that the IgA anti-VCA response is very sensitive to NPC, such that it is elicited whether the disease is clinical or subclinical. Nonetheless, it is apparent that the IgA anti-VCA test cannot be used to evaluate the evolution of NPC in individual patients.

It seems possible that IgA antibodies against VCA may be produced locally in the region of the tumour in the nasopharynx. This is compatible with the presence of EBV genomes in NPC cells (Klein *et al.*, 1974) which may be activated by treatment with halogenated nucleotides to synthesize EBV antigens and viral particles (Glaser *et al.*, 1976; Trumper, Epstein and Giovanella, 1976). Local production of IgA anti-VCA may account for the high frequency of detection of these antibodies in saliva and throat washings from NPC patients (Ho *et al.*, 1977; Desgranges and de Thé, 1977). Some of these locally produced antibodies might also be expected to reach the systemic circulation. If this were to occur extensively relative to the systemically produced antibodies, a general lack of correlation between serum IgA anti-VCA titre and IgA protein concentration might be anticipated, because the level of the largely locally-derived antibodies in the serum is not expected to be related to the overall activity of the systemic IgA immune system as reflected in the serum IgA concentrations. Serum IgG anti-VCA on the other hand, may be largely produced systemically and may thus reflect the intensity of systemic stimulation by VCA. The apparent lack of correlation between titres of serum IgA and IgG anti-VCA is thus compatible with different sites of production of these antibodies. The local production of IgA and IgG antibodies has been further studied by us. The results to be reported in the future show that there is a frequent occurrence of IgA plasma cells in NPC biopsy specimens, while IgG plasma cells were detected less frequently.

In an earlier study of concentrated saliva from NPC patients, we reported that the IgA antibodies in the majority of these specimens lacked the secretory piece, and it was speculated that this might reflect their systemic origin (Ho

*et al.*, 1977). This is contrary to the results of similar studies by Desgrange and de Thé (1977) who found that, in most instances, secretory pieces were associated with the IgA antibodies in the throat washings from NPC patients. This discrepancy between the results of these studies could be possibly due to dissociation of the secretory pieces from the IgA molecules during concentration, or to a difference in the antisera against the secretory piece used in these studies (de Thé, personal communication). We are currently attempting to resolve this discrepancy.

The observed frequency of detection of IgA anti-VCA in the sibs of NPC falls between those of NPC patients and controls. It may be inferred from the one instance described earlier of a sib of an NPC patient who had detectable IgA anti-VCA before clinical onset of NPC, and from the persistence of this reactivity in the relapse-free NPC survivors after RT, that the apparently healthy sibs with detectable IgA anti-VCA may harbour subclinical NPC. On the other hand, NPC patients' siblings with detectable IgA anti-VCA showed the binomial distribution pattern expected for the distribution of an autosomal recessive trait in sibships of different sizes. This is consistent with a possible genetic involvement in the IgA antibody response to VCA (Thompson and Thompson, 1966). Whichever the reason for the high frequency of detection may be, it is important to carry out a detailed study of EBV serology among the sibs of NPC patients and this is being pursued.

This work was supported in parts by the World Health Foundation (Hong Kong), the Hong Kong Anti-Cancer Society and by a research contract from IARC, Lyon, France. We are grateful to Professor John Aitchinson for advice on statistics.

### REFERENCES

Desgranges, C. & de Thé, G. (1977) Presence of Epstein Barr Virus Specific IgA in Saliva of Nasopharyngeal Carcinoma Patients: Their Activity and Possible Clinical Value. *Int. Symp. Etiology and Control of Nasopharyngeal Carcinoma, Kyoto, Japan*, April 1977.

Gerber, P., Goldstein, L. I., Lucas, S., Nonoyama, M. & Perlin, E. (1972) Oral Excretion of Epstein–Barr Virus by Healthy Subjects and Patients with Infectious Mononucleosis. *Lancet*, ii. 988.

Glaser, R., de Thé, G., Lenoir, G. & Ho, J. H. C. (1976) Superinfection of Epithelial Nasopharyngeal Carcinoma Cells with Epstein–Barr Virus. *Proc. natn. Acad. Sci., U.S.A.*, **73**, 960.

Henle, G. & Henle, W. (1966) Immunofluorescence in Cells Derived from Burkitt's Lymphoma. *J. Bact.*, **91**, 1248.

Henle, G. & Henle, W. (1976) Epstein–Barr Virus Specific IgA Serum Antibodies as an Outstanding Feature of Nasopharyngeal Carcinoma. *Int. J. Cancer*, **17**, 1.

Henle, W., Henle, G., Zajac, B. A., Pearson, G., Waubke, R. & Scriba, M. (1970) Differential Reactivity of Human Sera with Early Antigens Induced by Epstein–Barr Virus. *Science, N.Y.*, **169**, 188.

Ho, H. C. (1970) The Natural History and Treatment of Nasopharyngeal Carcinoma (NPC). In *Oncology* 1970. Ed. R. Lee Clark *et al.* Chicago Year Book Medical Publishers. p. 1.

Ho, J. H. C. (1972) Current Knowledge of the Epidemiology of Nasopharyngeal Carcinoma—A Review. In *Oncogenesis and Herpesvirus*. Ed. P. M. Biggs, G. de Thé & L. N. Payne. Lyon: IARC Scientific Publication, **2**, 357.

Ho, H. C., Ng, M. H. & Kwan, H. C. (1977) IgA Antibodies to Epstein–Barr Viral Capsid Antigens in Saliva of Nasopharyngeal Carcinoma Patients. *Br. J. Cancer*, **35**, 888.

Ho, H. C., Ng, M. H., Kwan, H. C. & Chan, J. C. W. (1976) Epstein–Barr Virus Specific IgA and IgG Serum Antibodies in Nasopharyngeal Carcinoma. *Br. J. Cancer*, **34**, 656.

Klein, G., Giovanella, B., Lindahl, T., Fialkow, P. J., Singh, S. & Stehlin, J. (1974) Direct Evidence for the Presence of Epstein–Barr Virus DNA and Nuclear Antigen in Malignant Epithelial Cells from Patients with Anaplastic Carcinoma of the Nasopharynx. *Proc. natn. Acad. Sci., U.S.A.*, **71**, 4737.

Thompson, J. S. & Thompson, M. W. (1966) *Genetics in Medicine*. Philadelphia: Saunders.

Trumper, P. A., Epstein, M. A. & Giovanella, B. C. (1976) Activation *in vitro* by BuDR of a Productive EB Virus Infection in the Epithelial Cells of Nasopharyngeal Carcinoma. *Int. J. Cancer*, **17**, 578.

Wara, W. M., Wara, D. W., Philips, T. L. & Ammann, A. J. (1975) Elevated IgA in Carcinoma of the Nasopharynx. *Cancer, N.Y.*, **35**, 1313.